# Development and Service Evaluation of an *ad hoc* Virtual Arthroplasty Clinic during COVID-19: Experiences from Irish National Orthopaedic Hospital

**DOI:** 10.5704/MOJ.2207.006

**Published:** 2022-07

**Authors:** B Murphy, P Carroll, R Daly, A McLaverty, P Keogh, J O'Byrne, J Cashman

**Affiliations:** Department of Trauma and Orthopaedic Surgery, Cappagh National Orthopaedic Hospital, Dublin, Ireland

**Keywords:** virtual arthroplasty clinic, COVID-19, telemedicine, hip, knee

## Abstract

**Introduction:**

COVID-19 has had a significant impact on healthcare. It has forced orthopaedic surgeons to limit face-to-face patient contact. This resulted in the *ad hoc* creation of a virtual arthroplasty clinic (VAC) in Irish National Orthopaedic Hospital. We aimed to assess this new VAC and ascertain its effectiveness as an alternative to physical appointments during and following the pandemic.

**Materials and methods:**

Patients were followed-up in this VAC six weeks post-operatively. A service evaluation of this virtual arthroplasty clinic was carried out using a questionnaire created by the orthopaedic department.

**Results:**

A total of 30 patients requiring 6-week follow-up after the arrival of COVID-19 in Ireland were included. Average pre- and post-operative visual analogue scale score (VAS) was 8.1 and 2.3, respectively. Average pre- and postoperative Oxford hip and knee score was 19.1 and 39.2, respectively. Twenty-one patients (70%) were happy to have their six weeks post-operative e-outpatient consultation virtually. Twenty-six patients (86%) were happy with future virtual follow-up. Twenty-eight patients (93%) would be happy experiencing the whole process again. Eleven patients would be interested in having future joint replacement surgery, though ten of them (91%) stated COVID-19 would impact that decision.

**Conclusion:**

Most patients were happy to have their six-week appointment and future appointments virtually. Functional outcome scores had improved and pain scores had reduced at six-week follow-up, supporting the idea that virtual clinics are not inferior to physical clinics. Patients expressed concern about having a further joint replacement in the context of COVID-19.

## Introduction

COVID-19 and the ensuing pandemic is perhaps the most disruptive global event to occur in our lifetime^1,2^. Orthopaedic surgeons were not spared from this disruption. The waiting lists for elective procedures, namely hip and knee arthroplasties, have become lengthy. Joint registries from the United States estimated that if even 50% of “non-essential” arthroplasty work was cancelled, over 15,000 primary procedures and nearly 1,500 revision procedures would be cancelled per week^3^.

The need for social-distancing measures and the suspension of “non-essential” clinical activity meant that we had to devise an *ad hoc* method of following-up these patients. In the interest of patient safety and to limit the number of face-to-face interactions during a period where COVID-19 was becoming more endemic, we developed what is a new concept for our hospital and not yet standard practice in our country, the “virtual arthroplasty clinic”.

The concept of virtual fracture clinics has been around for several years^4^ with virtual arthroplasty clinics less widespread. Current trends would suggest that COVID-19 has pushed surgeons even further towards the “virtual” mode of follow-up^5,6^. The drive to reduce unnecessary outpatient visits was evident, even prior to the pandemic^7^. Patients themselves are satisfied with these changes as much as the clinicians^8^. The existing models for patient follow-up are expensive and time-consuming. Pilot studies, using a “virtual” clinic like ours, have yielded some promising results in terms of reduced costs and time9. Virtual fracture clinics have been shown to have an important role during the COVID-19 pandemic^10^ and we hoped to recreate some of that success and satisfaction with our initiative.

Opponents of virtual follow-up say that omission of important clinical details is likely where a physical encounter is absent. However, pain and function scores like those used in our study have been shown to be able to detect risk of revision in certain patients^11^ without the need for physical examination.

The aim of this study was to develop a virtual arthroplasty clinic and then qualitatively assess it, hoping to create a new service that would remain relevant and safe post-COVID19 and thus have a lasting impact on hip and knee arthroplasty patient follow-up.

## Materials and Methods

We initiated a virtual arthroplasty clinic due to ongoing COVID-19 restrictions. Face-to-face appointments were cancelled, and patients were followed-up by telephone. This was an audio-only clinic, no video was used. It was a consultant-led clinic with assistance from the orthopaedic surgery trainees. This clinic functioned 6-weeks postoperatively, as this would be standard practice for normal physical appointments. The study aimed to qualitatively evaluate this new service. These virtual arthroplasty clinics included patients who had undergone either hip or knee arthroplasty. This service evaluation was conducted during the month of March 2020. A 14-point questionnaire was created by the orthopaedic department in our National Orthopaedic Hospital (Table I). A total of 3,364 combined hip and knee replacements had been performed in this hospital in 2019. The hospital deals with local referrals in the surrounding area but also with complex arthroplasties from around the country in a tertiary referral manner. It was renamed and rebranded as the Irish National Orthopaedic Hospital as a result. This questionnaire was used by orthopaedic surgery trainees in this hospital to evaluate the new service. Patients were asked to complete the Oxford Hip or Knee Score (where appropriate) and reported their scores as pre- and post-surgery. Patients were also asked to rank their pain pre- and post-operatively on a Visual Analogue Scale (0 being no pain and 10 being unbearable pain).

**Table I:** Results from service evaluation questionnaire, Oxford hip and knee scores and VAS

	n	Yes	No	Do not know	Min	Max	Mean
Age	30				46	80	67.0
Were you contacted by a member of the team after your joint replacement?	30	30 (100%)	0 (0%)				
How would you rate the experience out of 10 – 1 being a bad experience and	30				3	10	8.8
10 being an excellent experience?							
Was there anything you were satisfied or unsatisfied with?	30	8 (26.7%)	22 (73.3%)				
Would you have preferred to come to the outpatient department for follow-up?	30	9 (30%)	21 (70%)				
Have you had any problems with your joint replacement?	30	10 (33.3%)	20 (66.7%)				
Have you had previous joint replacement surgery?	30	13 (43.3%)	17 (56.7%)				
Considering COVID-19, we may need to follow-up joint replacement patients over	30	26 (86.7%)	4 (13.3%)				
the phone, like we have with you, moving forward – do you think this is a suitable							
method of follow-up?							
If you could go back in time, would you be happy to undergo joint replacement	30	28 (93.3%)	1 (3.3%)	1 (3.3%)			
surgery again?							
Are you planning on having another joint replacement in the future?	30	11 (36.7%)	18 (60%)	1 (3.3%)			
Would COVID-19 impact your decision to have further joint replacement surgery?	30	10 (33.3%)	18 (60%)	2 (6.7%)			
Oxford score pre-operative	29				6.0	36.0	19.1
Oxford score post-operative	30				9.0	48.0	39.2
VAS score pre-operative	30				2.0	10.0	8.1
VAS score post-operative	30				0.0	10.0	2.3

Patient data were collected and stored in a password-protected computer within the hospital. The office containing the computer was locked after office hours. The excel sheet containing the data was also password protected. Data collected included patient identification number, gender, occupation, the joint replaced and date of surgery. All patient data remained on the hospital computer for the duration of the study and were deleted after the study period had ended. Only the authors of this study had access to the data, as stipulated by the Research Ethics Board in this hospital.

## Results

Thirty patients were recruited into this service evaluation. These patients were chosen as they were originally to be followed-up face-to-face, but elective orthopaedic clinics were cancelled, coinciding with the first wave of COVID-19 to hit Ireland in March 2020. Fifteen of these patients had undergone knee arthroplasty and the other 15 patients hip arthroplasty. The average age of the knee and hip replacement patients was 67.7 years and 66.4 years, respectively. There were 18 male patients and 12 female patients. These procedures had been carried out by three consultant trauma and orthopaedic surgeons (n=14, n=13 and n=3 patients, respectively). Twenty-seven patients had primary procedures. In the knee replacement cohort, one patient had a unicompartmental knee replacement. There were three revision procedures (TKR n=2, THR n=1).

All procedures were carried out on dates between 21st January 2020 and 16th March 2020 in the national orthopaedic hospital.

Twenty-one patients (70%) were happy to have their 6-week post-operative outpatient consultation over the telephone and nine patients (30%) would have preferred to come personally to the clinic. However, 26 patients (86.7%) agreed that, considering ongoing COVID-19 restrictions, this method of follow-up for joint replacements was appropriate.

Twenty patients (66.7%) reported no problems with their joint replacement. When asked if they could go back in time, would they still have their joint replaced, 28 (93.3%) patients said they would. Seventeen patients (56.7%) had never had a joint replacement before this operation. Eighteen patients (60%) were not intending on having another joint replacement in the future. Eleven patients (36.7%) said they were planning on it and one patient (3.3%) was not sure. A total of 60% of patients (n=18) said that COVID-19 would have no impact on their decision to have another joint replacement in the future.

**Fig 1:**
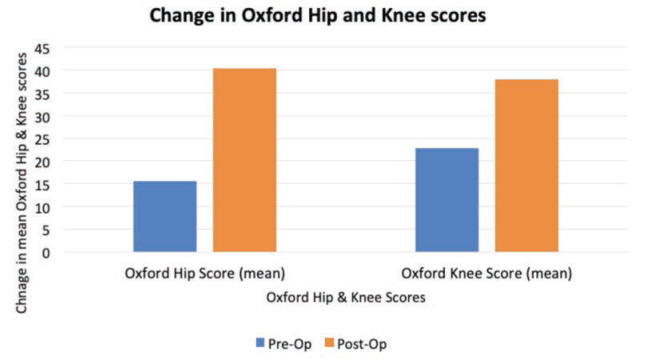
Mean Oxford hip and knee scores pre- and post-operative.

The mean pre-op Oxford knee score was 22.8. This increased to 38.0 post-operative. The mean pre-operative Oxford hip score was 15.6. This increased to 40.4 post-operative (Fig. 1).

**Fig 2:**
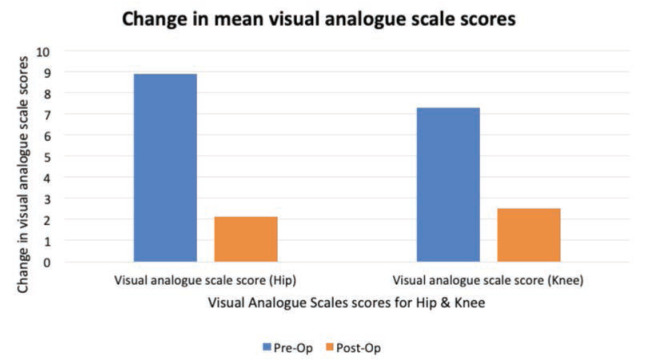
Mean visual analogue scale scores pre- and post-operatively.

In the knee replacement cohort, the mean pre-op VAS score was 7.3. This decreased to 2.5 post-operative. In the hip replacement cohort, the mean pre-op VAS score was 8.9. This decreased to 2.1 post-operative (Fig. 2).

On average, patients in the knee cohort rated the entire patient experience an 8.5/10 and the hip cohort rated it 9.1/10.

**Fig 3:**
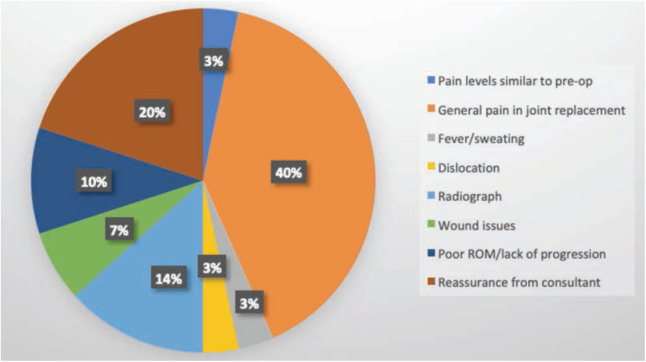
Pie chart highlighting issues that patients feel would need to be addressed in personal consultation.

Triggers for wanting to attend the outpatient department in person were explored in the questionnaire (Fig. 3). These included: pain levels similar to pre-op (n=1), pain in general in the replaced joint (n=12), fever or sweating (n=1), dislocation (n=1), a check radiograph for reassurance (n=4), wound issues (n=2), poor range of motion/lack of progression (n=3) and reassurance from consultant (n=6). The detailed results for each question asked are presented in Table II.

**Table II:** Results for each question asked as part of service evaluation

	n	%	Average age	Average rated experience	Average Oxford Pre-operative	Average Oxford Post-operative	Average VAS Pre-operative	Average VAS Post-operative
Location of injury								
Knee	15	50	67.7	8.5	22.8	38.0	7.3	2.5
Hip	15	50	66.4	9.1	15.6	40.4	8.9	2.1
Were you contacted by a member of the team after your joint replacement surgery?								
Yes	30	100	67.0	8.8	19.1	39.2	8.1	2.3
No	0	0						
Was there anything you were dissatisfied with?								
Yes	8	26.7	67.1	8.1	14.8	32.9	8.3	4.2
No	22	73.3	67.0	9.0	20.7	41.5	8.0	1.6
Would you have preferred to come to the outpatient department?								
Yes	9	30	65.0	8.1	20.0	34.0	8.0	2.8
No	21	70	67.9	9.0	18.7	41.4	8.1	2.1
Have you had any problems with your joint replacement?								
Yes	10	33.3	65.3	8.5	16.9	31.6	8.0	4.8
No	20	66.7	67.9	8.9	20.1	43.0	8.1	1.0
Have you had previous joint replacement surgery?								
Yes	13	43.3	71.1	9.2	18.4	39.0	8.4	3.1
No	17	56.7	63.9	8.5	19.5	39.4	7.8	1.7
Considering COVID-19, we may need to follow-up joint replacement patients over the phone, like we have with you, going forward – do you think this is a suitable method of follow-up?								
Yes	26	86.7	67.2	9.0	19.6	40.5	8.2	2.1
No	4	13.3	65.8	7.3	15.5	30.8	7.3	3.8
If you could go back in time, would you be happy to undergo joint replacement surgery again?								
Yes	28	93.3	66.7	8.9	19.4	40.1	8.3	1.9
No	1	3.3	68.0	5.0	10.0	14.0	6.0	9.5
Do not know	1	3.3	75.0	10.0		40.0	4.0	6.0
Are you planning on having another joint replacement in the future?								
Yes	11	36.7	65.3	8.3	15.4	36.4	8.5	3.4
No	18	60	67.4	9.0	21.3	41.1	7.9	1.6
Do not know	1	3.3	80.0	10.0	22.0	36.0	6.0	2.0
Would COVID-19 impact your decision to have further joint replacement surgery?								
Yes	10	33.3	64.5	8.9	19.0	42.8	7.7	0.7
No	18	60	68.5	8.7	19.8	37.3	8.5	2.8
Do not know	2	6.7	66.5	9.0	7.0	38.0	6.5	6.0

## Discussion

Our primary goal with the creation of a virtual arthroplasty clinic was to limit face-to-face interactions in our outpatient department to help prevent the transmission of COVID-19. Virtual follow-up reduces physical attendance without compromising patient safety with regards to the patient’s newly replaced joint^6,12^. It is important to remain cognisant of patient satisfaction levels when the traditional doctor-patient interaction has been altered, as is the case with these clinics. These virtual arthroplasty clinics are associated with a high patient satisfaction rate^13^ as was evident in our study where the majority (93%) would be happy to undergo the whole process again. An important aspect to note in our evaluation of this virtual arthroplasty clinic is that 70% of patients preferred this to face-to-face appointments. This would support its continued use in the future, thus reducing unnecessary visits to our outpatient department. Clinic waiting times would improve with the potential for positive financial implications in terms of money saved per patient, as has been the case with virtual fracture clinics^14,15^.

Some patients (30%) would have still preferred the traditional physical outpatient format as they found the virtual setting too impersonal and wished to be reassured in person. However, the majority (70%) were happy with the virtual format. This is a novel finding in our study, as recent evidence states there was no difference in patient satisfaction rates with telemedicine ventures such as our virtual clinic^16,17^. The findings of our study would suggest that we tailor these virtual clinics in the future to individual patient needs. They should be given the option of a face-to-face consultation if desired or better still, have a thorough explanation as to why they may not necessarily need to be seen in the outpatient department. This would help to alleviate anxiety and reduce unnecessary visits while also obtaining patient cooperation. In a time of crisis like the COVID-19 pandemic, using telemedicine ventures like this to our advantage allows for the continued care of patients while limiting unnecessary risks^18^.

In the present study, the patient cohort was less perturbed by COVID-19 than those seen in other studies. While other patient cohorts have been quoted as preferring to wait out the pandemic before having their surgery^19^, 60% of our patients said that COVID-19 would not impact their decision to have another joint replacement. This is a finding that policy makers may consider as this pandemic progresses. Patients waiting for elective hip and knee replacements pre-pandemic already found themselves on lengthy waiting lists^20^. Their ongoing pain and loss of function do not reduce during a pandemic. In fact, pain levels and analgesia have been shown to be higher in those patients whose operations have been cancelled compared to those who remain on a waiting list but have a date for surgery^21^. With the postponement of elective procedures many patients faced greater uncertainty regarding a potential new date for surgery. Our research would imply that we involve our patients in the decision-making process early in terms of whether to undergo surgery in the middle of a pandemic or not. Patients in this study were less likely to defer their arranged surgery as they perceived a lesser risk of contracting COVID-19 in hospital^22^.

The improvement in patients’ Oxford hip and knee scores and their VAS scores are encouraging findings. These scores are likely independent of outpatient follow-up and can be attributed to surgical intervention^23^. We must highlight that with our introduction of these virtual arthroplasty clinics, we are not changing the management of these patients, only their follow-up. The decision for surgical intervention (and the ensuing benefits) was not influenced. This may be reassuring and motivating for other hospitals considering the change to virtual clinics. It also re-affirms what we know about joint replacement surgery and the impact it can have on a patient’s quality of life^24^.

In terms of the triggers for wanting a face-to-face appointment, post-operative pain was the biggest factor. This is a useful finding as it will guide us in our future discussions with patients peri-operatively. It may be the case that our post-operative instructions regarding analgesia are not clear or detailed enough for patients. Our overall aim with this initiative and subsequent study was to reduce face-to-face encounters during the peak of COVID-19 and beyond if deemed acceptable by clinicians and patients. We have discussed satisfaction rates earlier and thus we should shift our focus towards mitigating these patient “triggers”. Further education regarding post-operative pain management has been a key theme in the literature for years and our study also highlights this^25^.

The free-text comment section (Table III) perhaps gave the greatest insight into patient attitudes and expectations which is in line with other studies^26^. In this initiative, patients were also keen for radiographs to confirm that they were progressing well and would have liked to receive that reassurance from a consultant orthopaedic surgeon rather than a junior doctor.

**Table III:** Qualitative patient comments regarding virtual arthroplasty clinic experience

Benefits	Drawbacks
Not having to attend hospital	Unable to relay concerns in person
Reduced risk if immunocompromised	Interaction feeling too impersonal
Convenience	Absence of physical examination
Opportunity to speak to a doctor with reassurance of a future appointment	Absence of radiograph for reassurance
Relaxed atmosphere	
Not feeling rushed during consultation	
Reassurance about pain levels and levels of progression	

There were some limitations associated with the present study. It was a relatively small sample of only 30 patients. However, this was a pilot study for this type of clinic in our institution and indeed Ireland. The procedures were carried out by three different orthopaedic consultants with the possibility that there may have been some variation in the subjective patient experience. Of the 30 procedures, three were revision surgeries. These are typically more challenging procedures for both the surgeon and the patient. Therefore, pain and function scores in these patients could feasibly have been affected. Knee range of motion in all patients was not measured formally due to the audio-only nature of the clinic. A disposable goniometer with ethical approval to make video calls would have improved this Aspect. This study was carried out at a time when COVID-19 had just emerged in our country, and it is possible that patients’ perceptions and attitudes towards the disease may have changed dramatically over the period of nearly two years that we are living with it.

## Conclusions

The introduction of a virtual arthroplasty clinic in our centre was a success. Most patients were happy with the format and would support its continued use in the light of a global pandemic. Most patients, having experienced the whole process and associated conditions, would be happy to have a joint replacement surgery again. Patients waiting for elective joint arthroplasty are not concerned about COVID-19 and are keen to alleviate their pain and improve their quality of life. There were objective improvements in patient pain and function scores using this new virtual format. Virtual arthroplasty clinics in the setting of COVID-19 present a reliable alternative to traditional method of arthroplasty follow-up, without compromising patient safety or satisfaction and in effect enhancing them. The initiative is an example of a successful pilot study that other centres may use as a standard follow-up strategy for arthroplasty patients.
